# Perceptions and practices surrounding the perioperative management of frail emergency surgery patients: a WSES-endorsed cross-sectional qualitative survey

**DOI:** 10.1186/s13017-022-00471-7

**Published:** 2023-01-18

**Authors:** Mallaika Viswanath, Darja Clinch, Marco Ceresoli, Jugdeep Dhesi, Mario D’Oria, Belinda De Simone, Mauro Podda, Salomone Di Saverio, Federico Coccolini, Massimo Sartelli, Fausto Catena, Ernest Moore, Deepa Rangar, Walter L. Biffl, Dimitrios Damaskos

**Affiliations:** 1 University of Edinburgh, Edinburgh, UK; 2 Registrar in General Surgery, Royal Infirmary of Edinburgh, Edinburgh, UK; 3 General and Emergency Surgery, School of Medicine and Surgery, Milano-Bicocca University, Monza, Italy; 4 Department of Ageing and Health, Guy’s and St Thomas NHS Foundation Trust, London, UK; 5 Division of Vascular and Endovascular Surgery, Cardiovascular Departments, University Hospital of Trieste, ASUGI, Trieste, Italy; 6 Unit of Digestive and Bariatric Surgery, Clinique Saint Louis, Poissy, Île-de-France France; 7 Emergency Surgery Unit, Department of Surgical Science, University of Cagliari, Cagliari, Italy; 8 Hospital of San Benedetto del Tronto, AV5 ASUR Marche, San Benedetto del Tronto, Italy; 9 Emergency and Trauma Surgery Department, Pisa University Hospital, Pisa, Italy; 10 Department of Surgery, Macerata Hospital, Macerata, Italy; 11 General and Emergency Surgery Dept, Bufalini Hospital, Cesena, Italy; 12 Denver Health System-Denver Health Medical Center, Denver, USA; 13 Medicine of the Elderly, Royal Infirmary of Edinburgh, Edinburgh, UK; 14 Scripps Memorial Hospital La Jolla, La Jolla, CA USA; 15 Department of General Surgery, Royal Infirmary of Edinburgh, Edinburgh, UK

**Keywords:** Frailty, Emergency surgery, Comprehensive geriatric assessment, Clinical frailty score

## Abstract

**Background:**

Frailty is associated with poor post-operative outcomes in emergency surgical patients. Shared multidisciplinary models have been developed to provide a holistic, reactive model of care to improve outcomes for older people living with frailty. We aimed to describe current perioperative practices, and surgeons’ awareness and perception of perioperative frailty management, and barriers to its implementation.

**Methods:**

A qualitative cross-sectional survey was sent via the World Society of Emergency Surgery e-letter to their members. Responses were analysed using descriptive statistics and reported by themes: risk scoring systems, frailty awareness and assessment and barriers to implementation.

**Result:**

Of 168/1000 respondents, 38% were aware of the terms “Perioperative medicine for older people undergoing surgery” (POPS) and Comprehensive Geriatric Assessment (CGA). 66.6% of respondents assessed perioperative risk, with 45.2% using the American Society of Anaesthesiologists Physical Status Classification System (ASA-PS). 77.8% of respondents mostly agreed or agreed with the statement that they routinely conducted medical comorbidity management, and pain and falls risk assessment during emergency surgical admissions. Although 98.2% of respondents agreed that frailty was important, only 2.4% performed CGA and 1.2% used a specific frailty screening tool. Clinical frailty score was the most commonly used tool by those who did. Screening was usually conducted by surgical trainees. Key barriers included a lack of knowledge about frailty assessment, a lack of clarity on who should be responsible for frailty screening, and a lack of trained staff.

**Conclusions:**

Our study highlights the ubiquitous lack of awareness regarding frailty assessment and the POPS model of care. More training and clear guidelines on frailty scoring, alongside support by multidisciplinary teams, may reduce the burden on surgical trainees, potentially improving rates of appropriate frailty assessment and management of the frailty syndrome in emergency surgical patients.

**Supplementary Information:**

The online version contains supplementary material available at 10.1186/s13017-022-00471-7.

## Introduction

Surgery is increasingly undertaken in an older population, with 5 million of the 11 million operations performed in the UK in 2016 occurring in those aged over 65 years [1]. In Australia, a 2014 report showed that women over the age of 85 comprised the largest proportion of emergency surgical admissions [2, 3]. Frailty is defined as “a condition characterised by loss of biological reserve, failure of physiological mechanisms and vulnerability to a range of adverse outcomes including increased risk of morbidity, mortality and loss of independence in the perioperative period” [4]. Frailty is recognised in approximately 17–30% of patients above the age of 65 undergoing emergency laparotomies [5, 6], and a higher frailty score (independent of age) is associated with adverse post-operative outcomes, including increased length of stay and mortality [7]. Frailty is also associated with higher rates of delirium, falls and infections [3, 4, 8, 9], all important clinical events that can complicate the admission of older surgical patients. The prevalence of frailty, and the association with adverse outcomes, highlights the importance of assessing and optimising the frailty syndrome, whenever clinically feasible, to improve post-operative outcomes.

The relatively new field of perioperative medicine has been proliferating as hospitals begin to consider multi-specialist management of frail, older surgical patients [10]. Different models have emerged, such as the orthogeriatric services. In Britain, the Guys and St Thomas NHS Trust started to develop the “Perioperative medicine for older people undergoing surgery” (POPS) model in 2003 [9]. This revolutionary model provides a geriatrician-led service using Comprehensive Geriatric Assessment (CGA) and optimisation methodology delivered by a multidisciplinary team, with the involvement of primary and community care, anaesthetic, and surgical services. By effectively assessing and optimising individualised care at a single point for complex older patients undergoing surgery, the POPS model improves outcomes in the perioperative period, leading to a reduction in post-operative morbidity and mortality [4, 9]. However, a 2019 survey showed that less than a quarter of NHS trusts have implemented the model and integrated whole-pathway geriatric medicine across surgical specialties [8]. Though this has improved since the initial 2014 study where only three trusts had integrated POPS [8, 11], it highlights the inequity of available services. The 2021 National Emergency Laparotomy Audit (NELA) reported that only 27.1% of frail patients over 65 undergoing an emergency laparotomy in the UK were reviewed by a geriatrician [12]. It is nevertheless an improvement from the 2015 report where this number was about 10% [13]. Some of the existing barriers in the UK include a lack of funding and knowledge about perioperative care as well as a lack of inter-specialty coordination, which is vital to establishing the POPS model [11].

An alternative model of care, the hospitalist approach, entails the co-management of surgical patients by a surgeon and hospitalist such as a specially trained anaesthesiologist [14] or physician who is dedicated to the general medical care of hospitalised patients [15]. This model has been successful in the USA and Italy, where it has reduced the length of patient stay and readmissions, as well as resulted in significant financial saving for hospitals which implemented it [14, 15].

The British Geriatrics Society and Centre for Perioperative Care recently published guidelines [4] detailing the need for frailty assessment and how a multidisciplinary approach to perioperative management of frail patients undergoing surgery can be delivered. However, the reach of such guidelines is not known, despite surgical trainees reporting that they feel inadequately prepared or supported to manage complex older surgical patients [16]. We conducted this survey to gain insight into the real-life application of the guidelines and recommendations of various organisations and societies regarding the importance of perioperative risk scoring and frailty. We hypothesise that frailty is under-recognised worldwide and that multidisciplinary care of frail emergency surgery patients is not widespread.

This study aims to determine:

1. What are the current perioperative practices for frail patients undergoing emergency surgery and how are they implemented worldwide?
2. What are surgeons’ opinions and perceptions about frailty and its impact on perioperative outcomes?
3. What are the key barriers to implementing comprehensive perioperative management for these high-risk patients?

## Methodology

A qualitative cross-sectional survey was created on Google Forms and reviewed by a consultant general surgeon and the WSES Board. Information about the study and a link to the electronic survey were emailed to WSES members via a weekly e-letter. The survey was closed after 8 weeks (21.12.2021–15.02.2022). Participation was voluntary and consent was implied through the completion of the survey. No incentives were offered for participation.

The self-administered questionnaire contained a mixture of multiple-choice and 5-point Likert-style questions. Some questions allowed participants to select multiple responses and provide free text answers. It was divided into three domains:

1. Demographic details
2. Frailty assessment and risk stratification in emergency surgery patients
3. Surgeons' awareness of perioperative medicine, current perioperative care practices and barriers to implementation.

The full survey can be accessed here: Additional file 1.

Responses were analysed using descriptive statistics and reported by themes. The themes were: risk scoring systems, frailty awareness and assessment, and barriers to implementing practice. Respondents were invited to mark all options which applied, and therefore the results may add up to over 100% in some questions. Responses were grouped into academic and non-academic hospitals for analysis.

Categorical data were analysed as percentage bar charts. All analyses were carried out using Microsoft Excel.

## Results

### Demographics

We had 168 respondents, out of 1000 active WSES members. Table 1 shows respondents’ characteristics. Most of our respondents were European (83.3%, *n* = 140), with a small proportion from Asia (7.7%, *n* = 13). General surgery was the most common specialty (86.9%, *n* = 146). Most respondents held consultant roles: 51.2% (*n* = 86) were senior consultants, 31.5% (*n* = 53) were junior consultants. A minority (14.9%, *n* = 25) of our respondents were trainees. 63.7% (*n* = 107) of respondents worked in an academic hospital.

**Table 1:** Respondent demographics

		N	% of respondents
Continent of practice	Asia Africa Europe North America South America Oceania	13 5 140 7 1 2	7.7% 3% 83.3% 4.2% 0.6% 1.2%
Specialty	General surgery Trauma surgery Orthopaedic surgery Vascular surgery GI surgery Anaesthesiology HPB, transplant Intensivist Breast surgeon	146 13 2 1 1 1 1 1 1	86.9% 7.7% 1.2% 0.6% 0.6% 0.6% 0.6% 0.6% 0.6%
Level of training	Senior consultant Junior consultant Trainee Other	86 53 25 4	51.2% 31.5% 14.9% 2.3%
Years of professional experience	0–5 6–15 > 15	38 67 63	22.6% 39.9% 37.5%
Type of hospital	Academic Non-academic: Public/state Rural Ambulatory surgery centre Private District general	107 61 53 4 0 3 1	63.7% 36.3% 31.5% 2.4% 0% 1.8% 0.6%

### Risk stratification practices

**Fig. 1:**
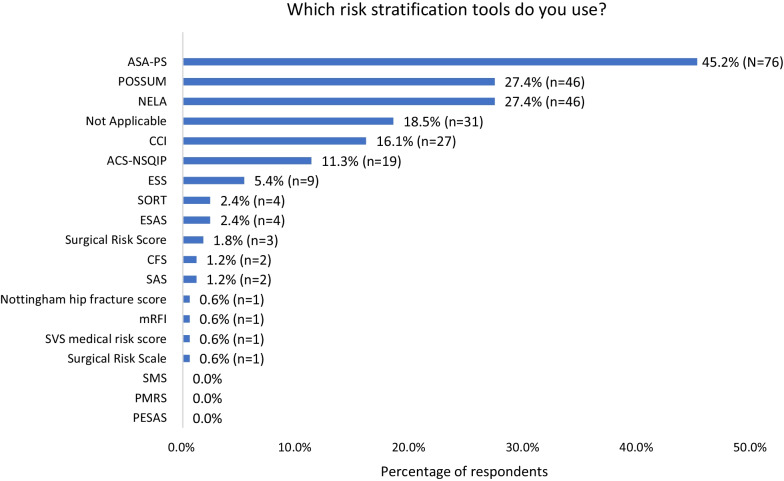
RSTs used by respondents. List of unabbreviated RSTs in Additional file 1

33.3% (*n* = 56) of participants said they did not risk score all patients using a validated Risk stratification tool (RST) prior to emergency surgery. Figure 1 shows which RSTs respondents used: the most used RSTs included ASA-PS, NELA and POSSUM. These did not vary by type of hospital (Additional file 1: Figure S1).

### POPS model and frailty

61.3% (*n* = 103) of our respondents were unaware of the POPS model and CGA, and this did not vary depending on whether they were in an academic hospital (60.4%) or not (62.3%). There was no meaningful variance by country (Additional file 1: Figure S2).

**Fig. 2:**
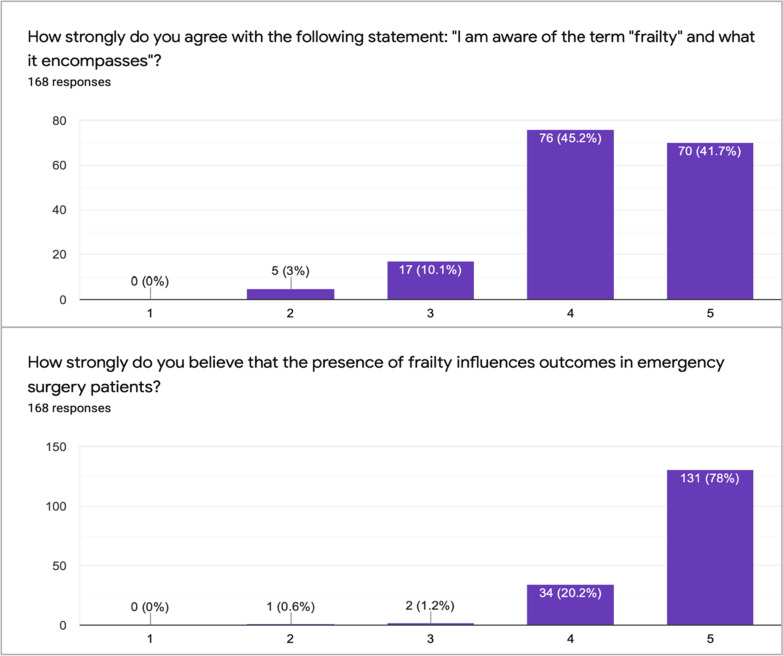
Likert scale questions on Frailty (1 = strongly disagree, 3 = neutral, 5 = strongly agree)

Figure 2 shows how strongly respondents agreed that they understood the term “frailty” and if they believed it influences outcomes in surgery. 86.9% (*n* = 146) agreed or strongly agreed that they understood frailty. 98.2% (*n* = 165) of respondents believed that the presence of frailty influences outcomes in emergency surgery patients.

### Frailty assessment

**Fig. 3:**
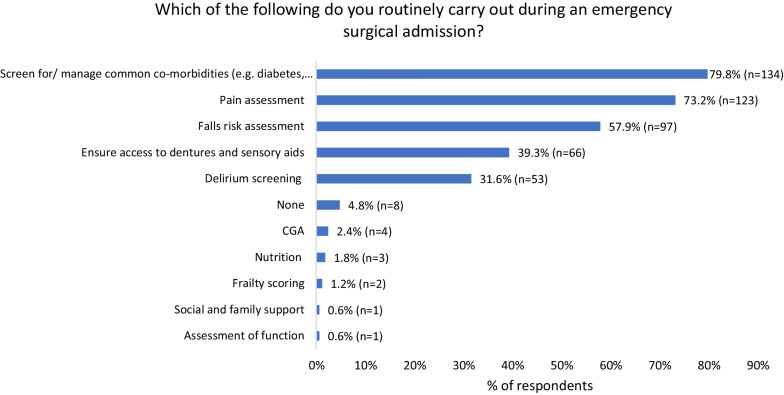
Routine pre-assessments conducted during emergency surgical admissions

Figure 3 shows which pre-surgical assessments are routinely carried out by respondents during an emergency surgical admission. Comorbidity management and pain assessment were most regularly done. 4.8% (*n* = 8) did none of the assessments routinely. Only 1 respondent routinely frailty scored, and 4 respondents (2.3%) used CGA through a geriatric liaison.

**Fig. 4:**
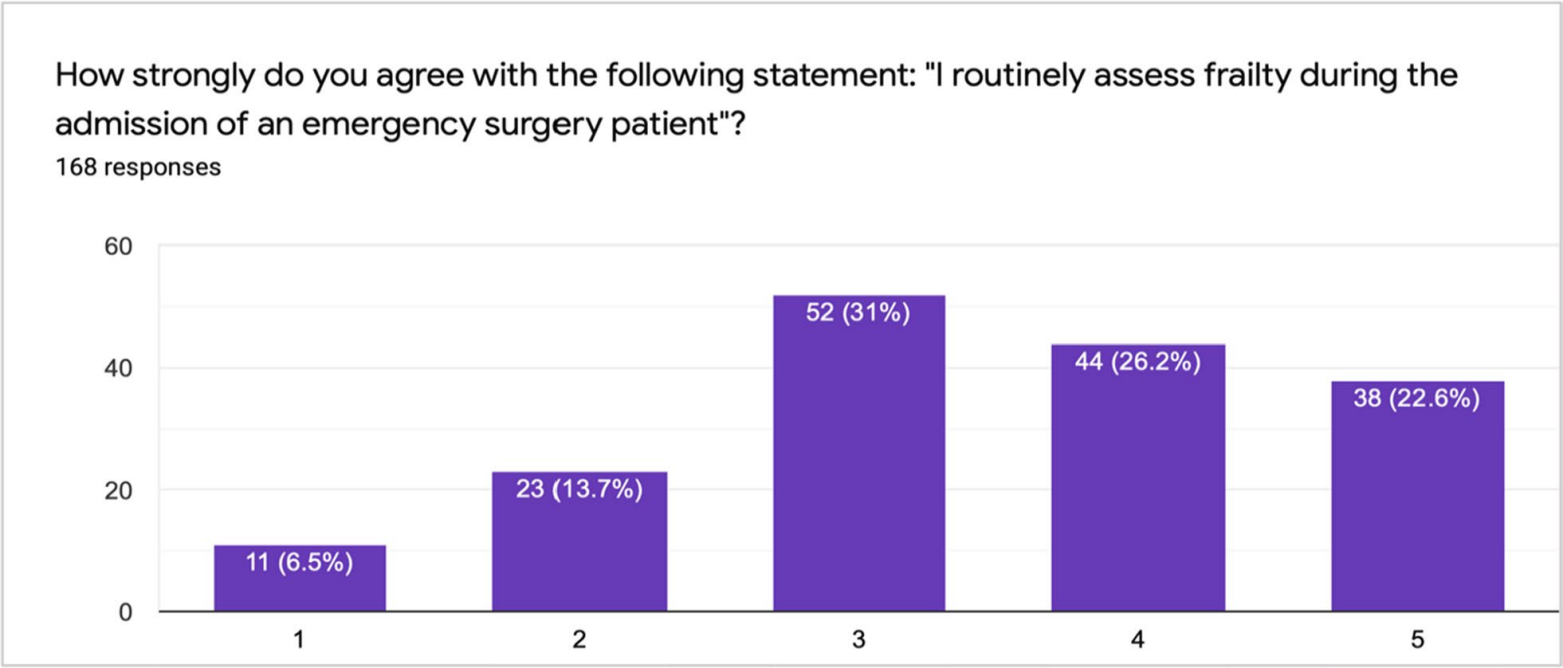
Likert scale of frailty scoring by patients (1 = strongly disagree, 3 = neutral, 5 = strongly agree)

**Fig. 5:**
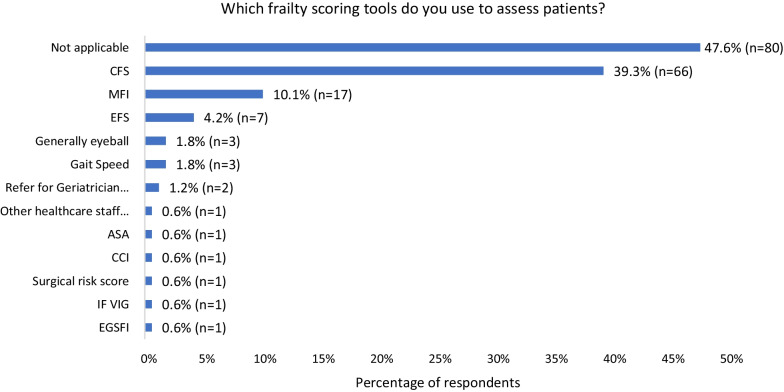
Clinical frailty scales used by respondents

There was a mixture of answers when asked whether respondents routinely frailty scored (Fig. 4), with the majority neither agreeing nor disagreeing with the statement. However, almost half (47.6%, *n* = 80) said the use of a formal scoring tool was not applicable and 3 participants reported they base it on general “eyeballing” (Fig. 5*)*. Clinical Frailty Score (CFS) was the most commonly used frailty tool, followed by the modified frailty index (MFI) (Fig. 5). When asked whose responsibility scoring is, participants responded that frailty scoring is mostly conducted by surgical trainees (44%, *n* = 74), followed by surgical nurses and consultants (29.8%, *n* = 50 and 28%, *n* = 47, respectively). Only 15.5% (*n* = 26) of respondents said geriatricians reviewed patients.

### Barriers to frailty assessment

**Fig. 6:**
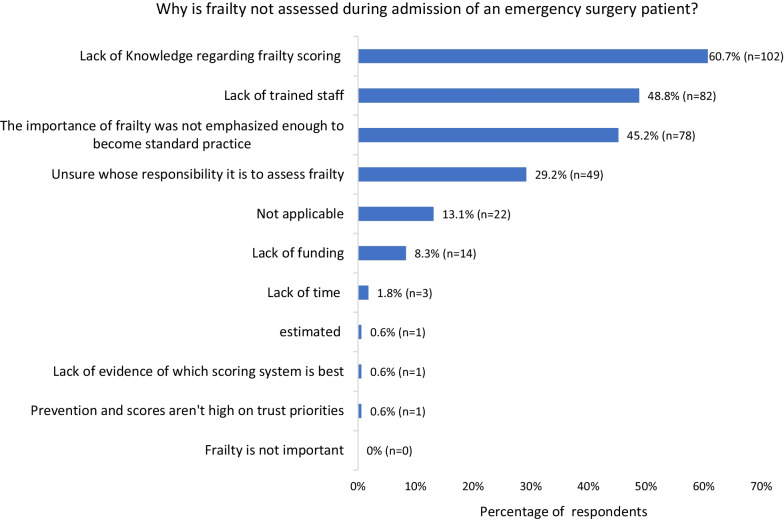
Common reasons why frailty was not assessed

We allowed participants to select from multiple common reasons why frailty was not assessed and had a free text option to suggest others. Thematic analysis revealed that lack of knowledge and training regarding clinical frailty scoring were key barriers. Participants reported not knowing about frailty scoring tools or why scoring is important. Other themes included not having enough time and being unsure whose responsibility scoring was. Only 8.3% (*n* = 14) of respondents stated lack of funding as being a barrier to scoring. Figure 6 shows the complete list of responses.

### Current perioperative care practices

Over half of our respondents had poor awareness of the term “perioperative physician” (mean Likert score 3.03). Anaesthesia was the specialty respondents most associated with that term (69.5%, *n* = 117), followed by surgery (53.6%, *n* = 90) and geriatrics (42.3%, *n* = 71). Only 12% (*n* = 20) of responses associated it with GP and 1 respondent put “all specialties” as a free text answer.

60.7% (*n* = 102) of respondents said that other specialties in their hospital have physician or geriatrician input, although only 25.6% (*n* = 43) said geriatrician input is routinely asked for in frail emergency surgery patients. Most (40.5%, *n* = 68) said they only seek physician/geriatrician review post-surgery to facilitate discharge, and 28% (*n* = 47) said that the surgical team manages all issues. A common theme seen is that physician review is only requested in select patients with complications or co-morbidities, but not routinely. When an input is requested, 81.5% (*n* = 97) of respondents said it was delivered on demand of the surgical team, and only 19.3% (*n* = 23) of respondents said it was delivered during routine ward rounds. Only 10.1% (*n* = 12) of respondents said geriatricians were embedded in the surgical team.

Amidst respondents who do ask for physician input (*n* = 118), three main themes emerged. The first was managing medical problems such as hypertension or pulmonary oedema (78.3%, *n* = 94), and medication review (56.7%, *n* = 68). Another theme was facilitating holistic patient care by discussing shared decision-making (33.3%, *n* = 40), “do not attempt resuscitation” (DNAR) forms (20.8%, *n* = 25), goals and expectation of care (40%, *n* = 48), and arranging rehab (48.3%, *n* = 58) or discharge (75.8%, *n* = 91). The third theme was seeking advice regarding issues of the elderly such as management of the frailty syndrome (51.7%, *n* = 65) and delirium (50.8%, *n* = 61).

**Fig. 7:**
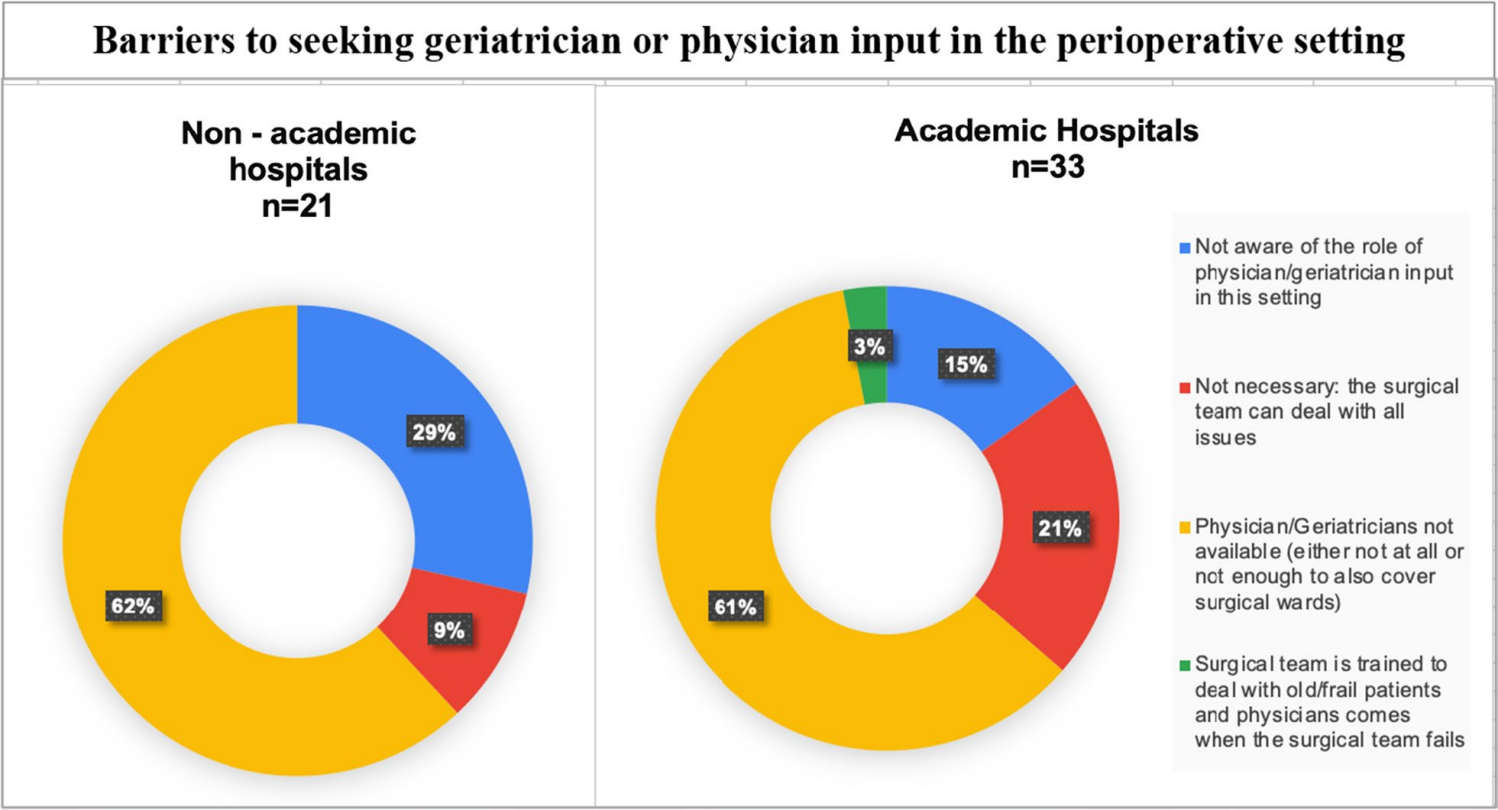
Pie chart showing reasons why participants did not seek geriatrician input in academic versus non-academic hospitals

Of those who reported that they did not seek physician input, lack of staff availability was cited as the key reason in both academic and non-academic hospitals (Fig. 7). Lack of knowledge about the role of physicians in perioperative medicine was the second-most common barrier in non-academic hospitals, whilst in academic hospitals more participants felt that all issues could be handled by the surgical team.

This survey successfully generated interest in perioperative medicine as 84.5% (*n* = 142) of participants agreed that they would consider reviewing relevant literature to find out more about the topic.

## Discussion

To our knowledge, this is the first study amongst emergency surgeons from around the world focusing on awareness of perioperative risk scoring and frailty, assessment of frailty and barriers to the multidisciplinary management of frail emergency surgical patients. It demonstrated that frailty is underassessed and undertreated despite a satisfactory level of awareness of the frailty syndrome, due to insufficient knowledge regarding perioperative frailty assessment and lack of trained staff. Models such as POPS are not universally recognised, and there is much uncertainty about the role of frailty scoring in an emergency setting.

Even though almost all respondents agreed that frailty influences outcomes in emergency surgery patients, only a small proportion routinely assessed it due to lack of knowledge or training on frailty scoring. It can sometimes be argued that frailty assessment cannot influence clinical practice in the emergency setting. However, we feel that patients who clearly are too frail for major interventions with anticipated poor outcomes may be considered for palliative/end-of-life/comfort care treatment instead of emergency surgery, as any major intervention would be unlikely to change the final outcome or have a meaningful impact on the patient’s quality of life. CFS was the most popular frailty scoring tool, perhaps because it is quick and easy to assess which lends itself easily to an urgent emergency setting. Nonetheless, it is hard to distinguish if it is used as recommended or if a gestalt assessment is made, subjective to the observing physician: three of our respondents admitted to “eyeballing” the patient. Others used inappropriate tools, such as RSTs when asked how they frailty score patients. This suggests there is poor awareness of the distinction between the tools and what they are informing, leading to inappropriate use in the perioperative setting. More knowledge on the condition of frailty and its consequences may help clarify the distinction between medical co-morbidities and frailty and how it informs our care, thus optimising outcomes for our elderly patients who likely bear the burden of both. The fact that there are so many scoring schemes further suggests they are not consistent or that the scientific evidence to justify their use is weak. Future research could assess the best scoring systems and clarify where they are useful, although national guidelines in the UK advocate using CFS as the initial screening tool in all settings.

Most of our respondents were unaware of the POPS model or CGA, with only 4 respondents using CGA. Although more respondents from Europe seemed to be aware of the models than in other continents, this may be attributed to the fact that most respondents were Europeans, as well as because both models are British and may not have circulated beyond Europe yet. We would expect there to be more awareness in Europe and in academic hospitals than in rural hospitals, particularly seeing as most respondents were consultant level. However, this lack of awareness even by seniors in these settings, suggests that the burden of frailty may be even more poorly managed in non-academic hospitals and developing countries.

Key barriers for those who did not assess frailty included staff being unsure of whose responsibility it is to do so and poor knowledge regarding validated frailty scoring tools. Providing clear guidelines about frailty scoring, what stage of admission and by whom it must be done, may help guide preoperative optimisation. This may be more beneficial if the frailty scoring translated directly into clinical management. The emergence of novel artificial intelligence (AI) systems may have a role to play in the identification of frailty [17, 18] and optimisation of multimorbid geriatric care [19] in the future. Using AI may help omit human bias and speed up holistic frailty management, which would be particularly useful in an emergency setting.

Only 66.6% of respondents use a validated RST and document the results on patients prior to emergency surgery. Tools such as ESAS and ACS-NSQIP are best to use in emergency general surgery [20], but showed poor uptake by our respondents. Guidelines such as those by ERAS (enhanced recovery after surgery) also suggest NELA and POSSUM as they are more likely to predict actual risk in emergency laparotomy patients [21]. Whilst they were also used, they are more time-consuming, which may explain why they are used significantly less than the more popular ASA [22].

Anaesthesia was still the specialty most associated with perioperative medicine, despite growing evidence that multispecialty care teams are more effective than any one specialty alone[8, 11]. Models such as POPS highlight the effectiveness of involving specialty geriatric input early in the management of frail surgical patients, although the role of a perioperative physician is still ill-defined. Despite surgical trainees being poorly educated on managing frail patients [16], our study found that only a quarter of respondents routinely ask for geriatrician input and that this is primarily done when there are complications or specific co-morbidities which require managing. This “reactive” model of care has been shown to be less effective than proactive management [11]. Very few respondents had a geriatrician embedded in their team, despite evidence that specialties such as orthogeriatrics show significantly improved outcomes post-hip fracture [23].

Despite post-operative geriatrician input having been shown to reduce patient mortality after emergency laparotomies [24] and reduce inpatient stay after GI surgery [25], we found that there are still barriers to implementing this into practice. A UK study suggested a lack of funding at an administrative level as a key barrier to organising this model of care and training in CGA [11]. The core barrier for our respondents was a lack of staffing, which combined with a lack of awareness of the role of geriatrician in an emergency surgical setting meant many were unable to involve geriatricians in the routine management of frail emergency patients. Most respondents said surgical trainees conducted frailty assessment highlighting the gap in providing surgical trainees with clear guidelines and ensuring they have sufficient support. This is in line with current attitudes of UK trainee surgeons who feel they are inadequately supported by geriatricians and feel they would benefit from shared management of patients (16). Recirculating this survey amongst trainees might give us a better idea of their knowledge and perceptions around perioperative frailty management.

A key limitation of this study is the low response rate; thus, it would be more statistically sound to say this is a subset of data and any inferences would not be representative of the whole population. Another limitation was that most respondents were from Europe, despite the survey being sent to all the members of WSES around the world. Repeating the survey in the future may mitigate this. However, the limited response may also indicate a lack of knowledge of this important topic amongst our target population deterring them from participating. There is scope for respondent bias due to participants being members of WSES and most respondents being from Europe and general surgery, which may reflect that perioperative medicine is of more interest in this region and specialty than others. Nonetheless, we would expect respondents who chose to participate due to specialist interest in this topic to be more aware of POPS and frailty than others; thus, the poor awareness highlighted by this study is worrying as it is likely to be pervasive throughout other regions and specialties in medicine. We also do not have data on the relative percentages of respondents per continent to the actual number of WSES members per continent for further analysis. Not all possible models of care have been evaluated in depth, but care was taken to include as many possible models as possible, based on the published literature. Future research could look at practices of surgeons in other specialities as a comparison.

Our results highlight the gap in translating perioperative frailty management guidance into routine clinical practice. The burden of perioperative management is not being sufficiently undertaken by emergency surgical teams and there is uncertainty around the perioperative management of frailty. More engagement on the role and benefits of the perioperative physician and its impact on patient outcomes may translate into future funding for training on CGA and to support the organisation of multispecialty surgical teams. Increasing awareness of existing clinical practice guidelines may encourage more hospitals worldwide to uptake this into routine practice.

In conclusion, we found that whilst most surgeons are aware of the importance of frailty in affecting surgical outcomes, there is poor awareness of the role of CGA, and the various models and guidance that positively influence the outcomes of high-risk and frail emergency surgical patients. Formal frailty assessment is not routinely done, and the key barriers to this seem to be lack of knowledge about frailty and assessment, lack of trained staff and uncertainty around whose responsibility it is. The establishment of multidisciplinary teams with geriatric input would eliminate these uncertainties and share the burden of perioperative management of frail emergency surgical patients to ensure better outcomes in the long term. We believe that risk scoring for emergency surgery and frailty assessment using universally validated tools are of paramount importance in the holistic treatment of emergency surgery patients and this assessment should be part of every emergency surgery admission documentation, for the multidisciplinary team (surgeon/anaesthetist/intensive care/geriatrician if applicable) to make the best possible decision for the patient and with the patient. This study will hopefully raise awareness and encourage participants to review the relevant literature, leading to the development of more comprehensive guidelines regarding frailty management in the emergency surgical setting.

## Supplementary Information

**Additional file 1.** S1. Full Google forms Survey. S2.1. Bar graph showing whether the use of Risk Stratification Tools varies by type of hospital. S2.2. Bar graph showing respondents’ awareness of the terms POPS and CGA, by country and type of hospital.

## Data Availability

The data sets used and/or analysed during the current study are available from the corresponding author upon reasonable request.
