# Supplementary material for: Perceptions and practices surrounding the perioperative management of frail emergency surgery patients: a WSES-endorsed cross-sectional qualitative survey
Source: World J Emerg Surg. 2023 Jan 18;18:7. doi: 10.1186/s13017-022-00471-7 (PMC9850554; doi:10.1186/s13017-022-00471-7)
Supplement: Supplementary file 1 — Additional file 1. S1. Full Google forms Survey. S2.1. Bar graph showing whether the use of Risk Stratification Tools varies by type of hospital. S2.2. Bar graph showing respondents’ awareness of the terms POPS and CGA, by country and type of hospital. [file 13017_2022_471_MOESM1_ESM.docx]

**Appendix (i) with survey**

**
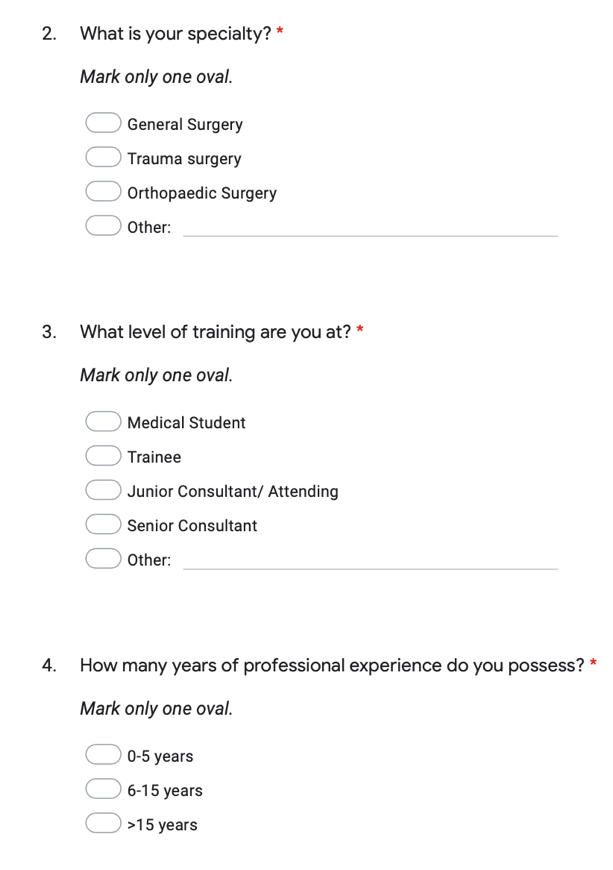
**

**
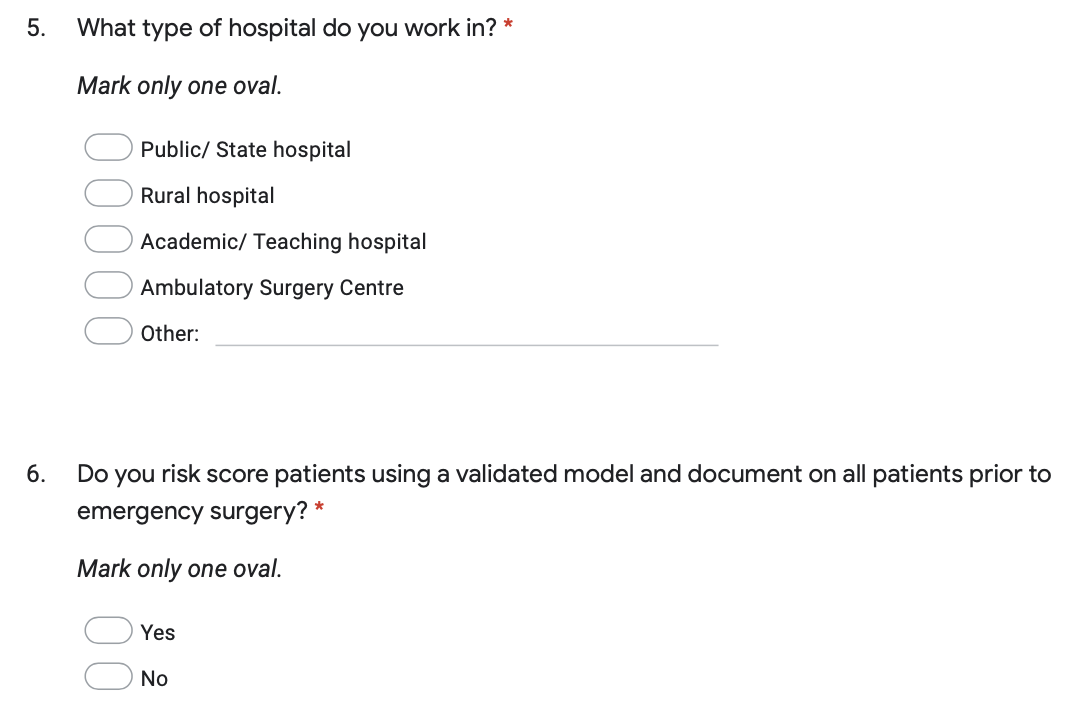

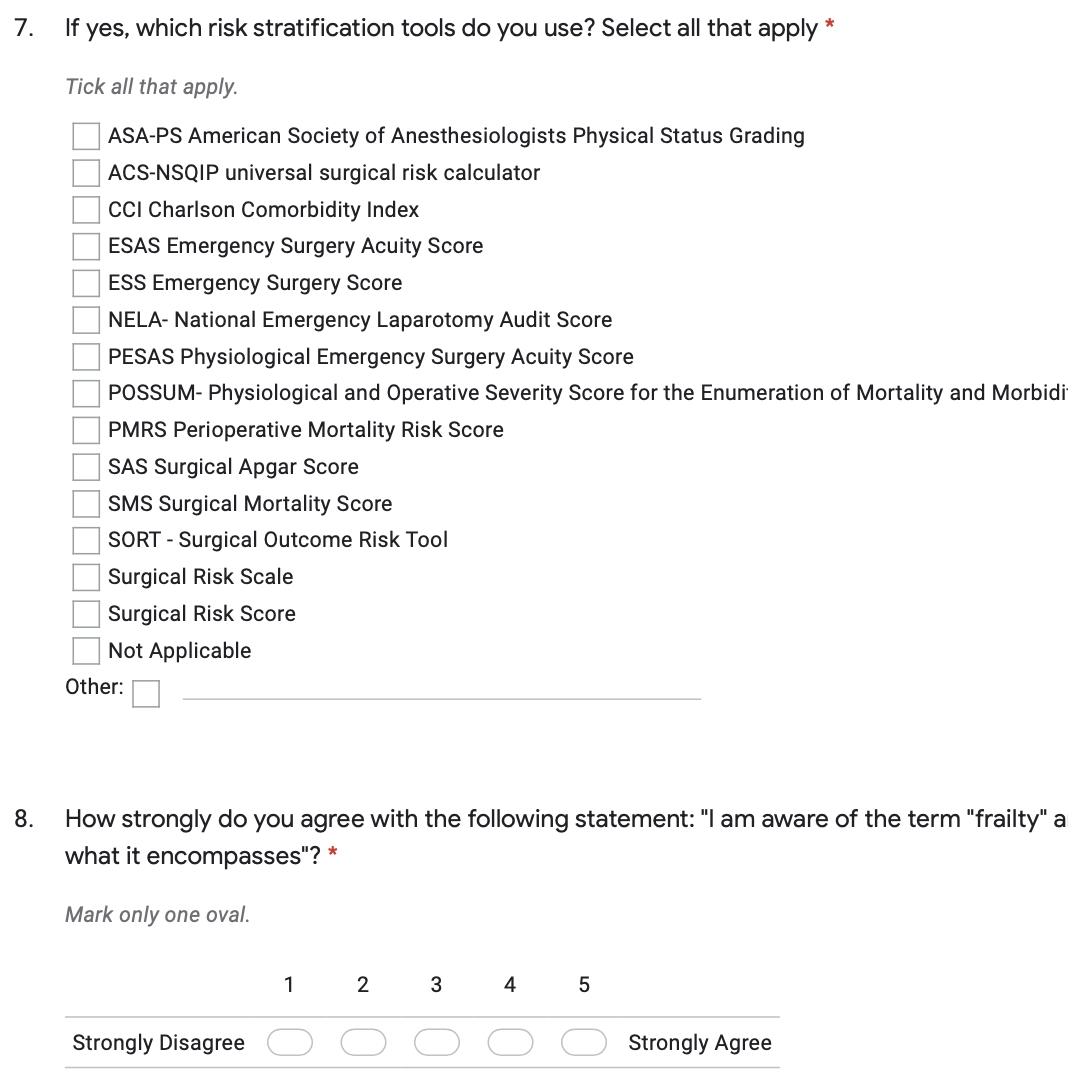

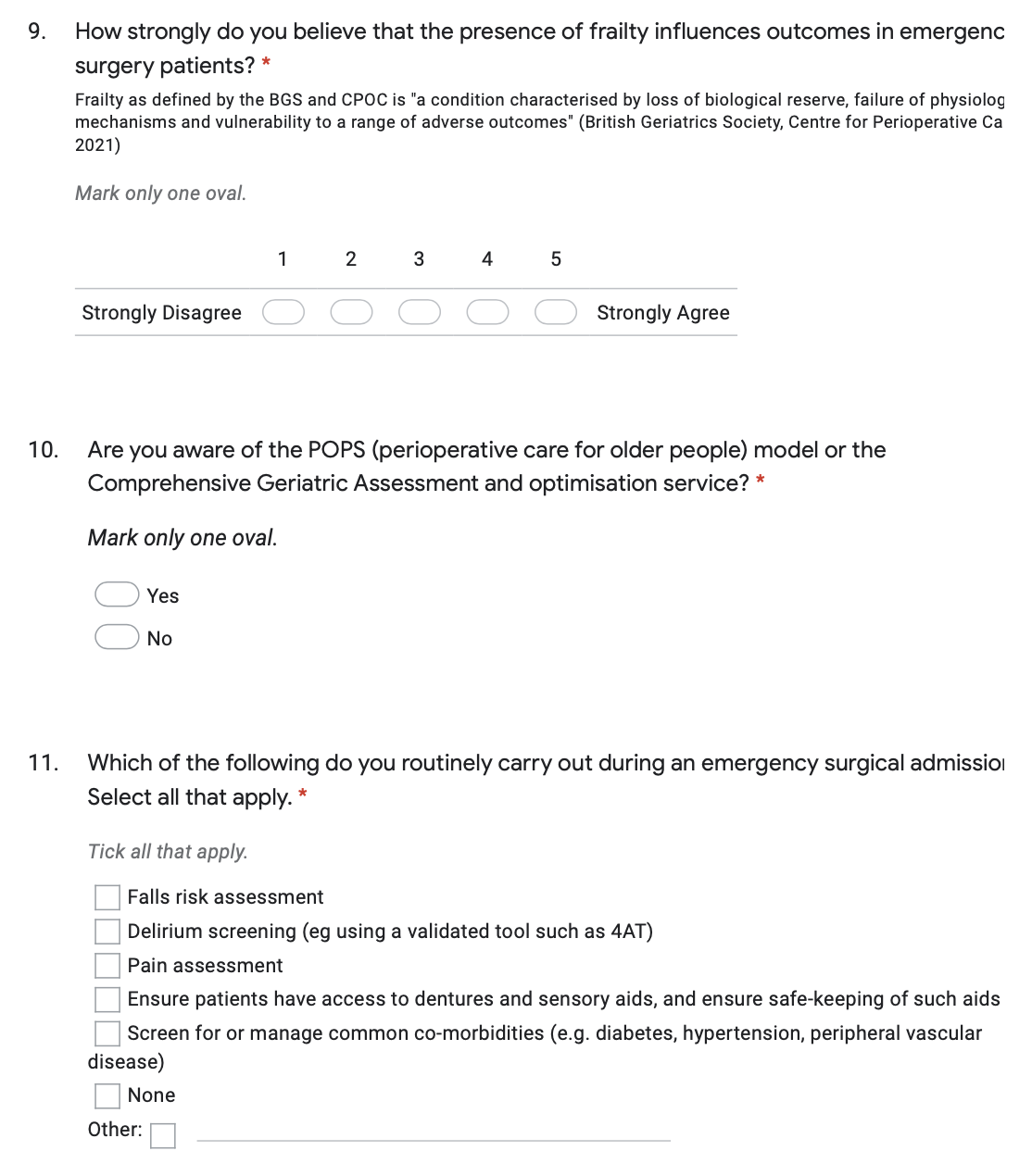

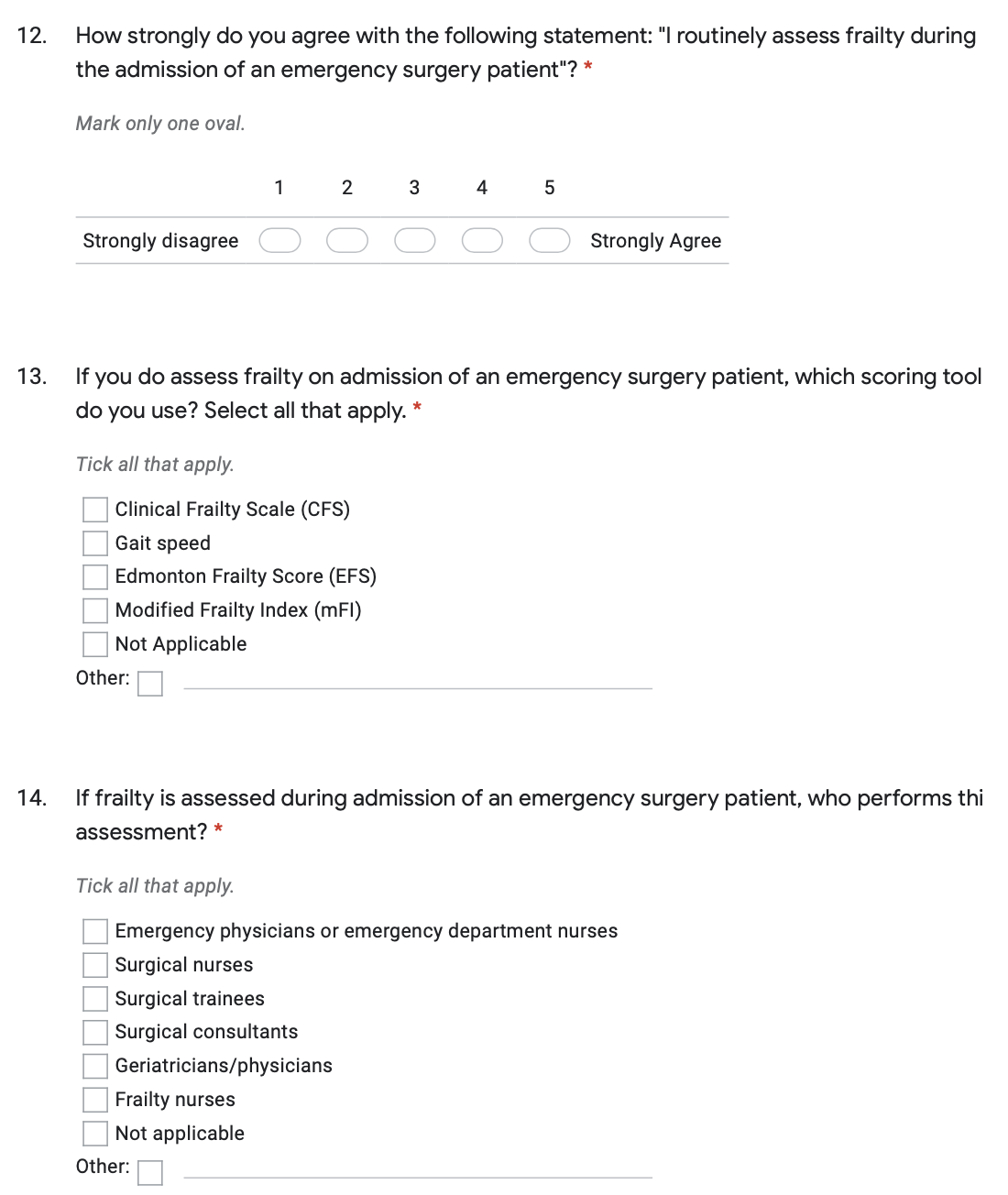

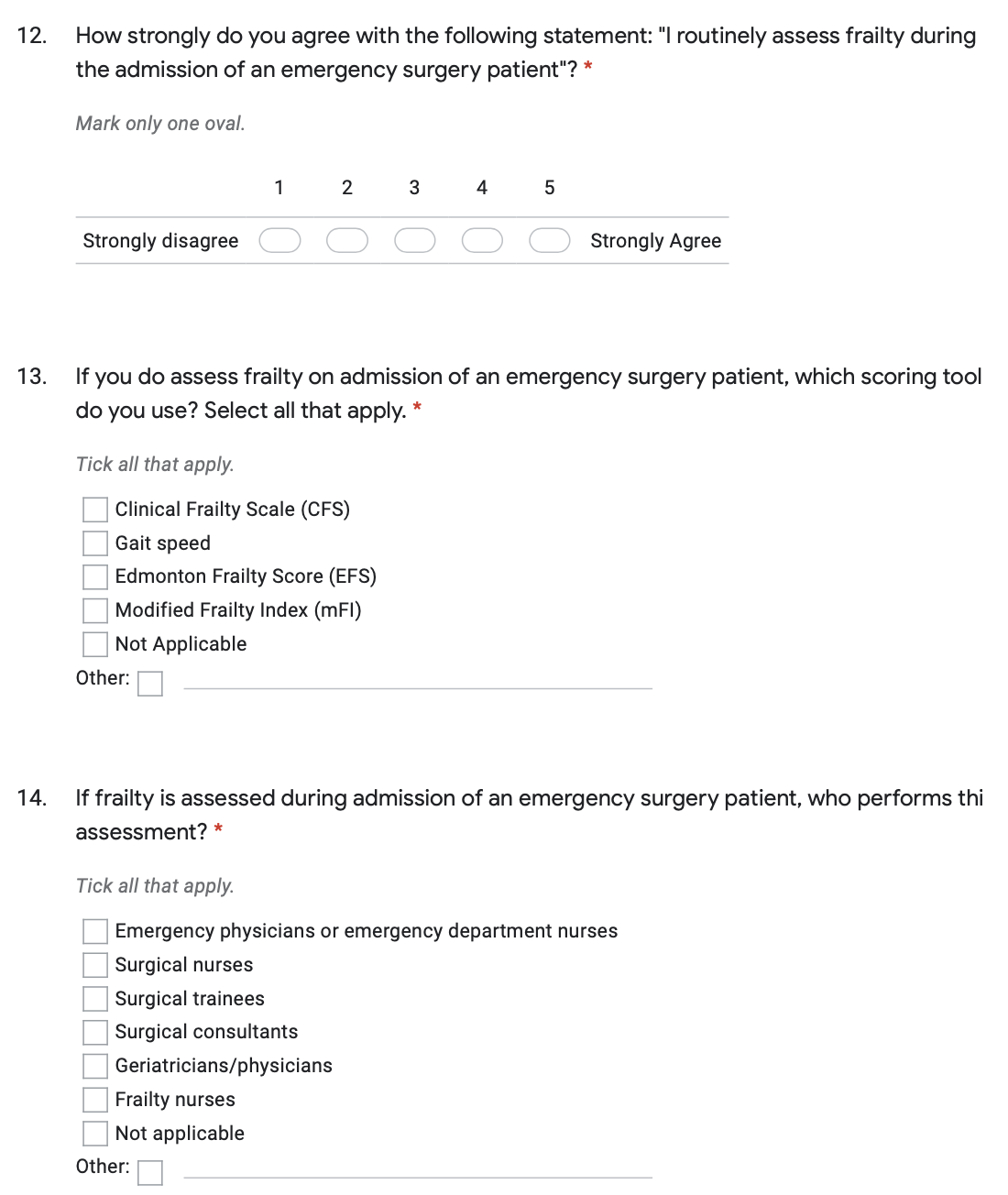

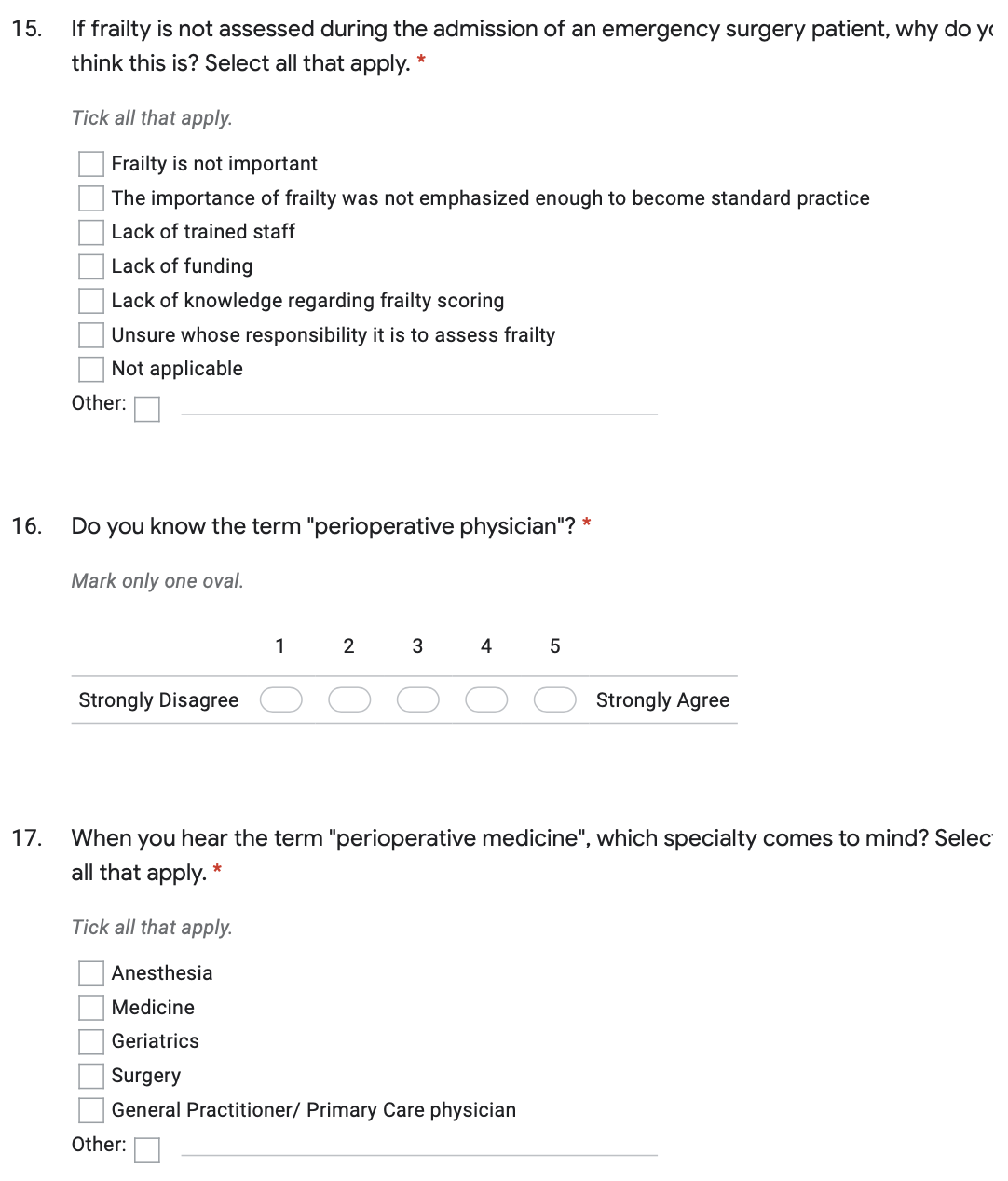

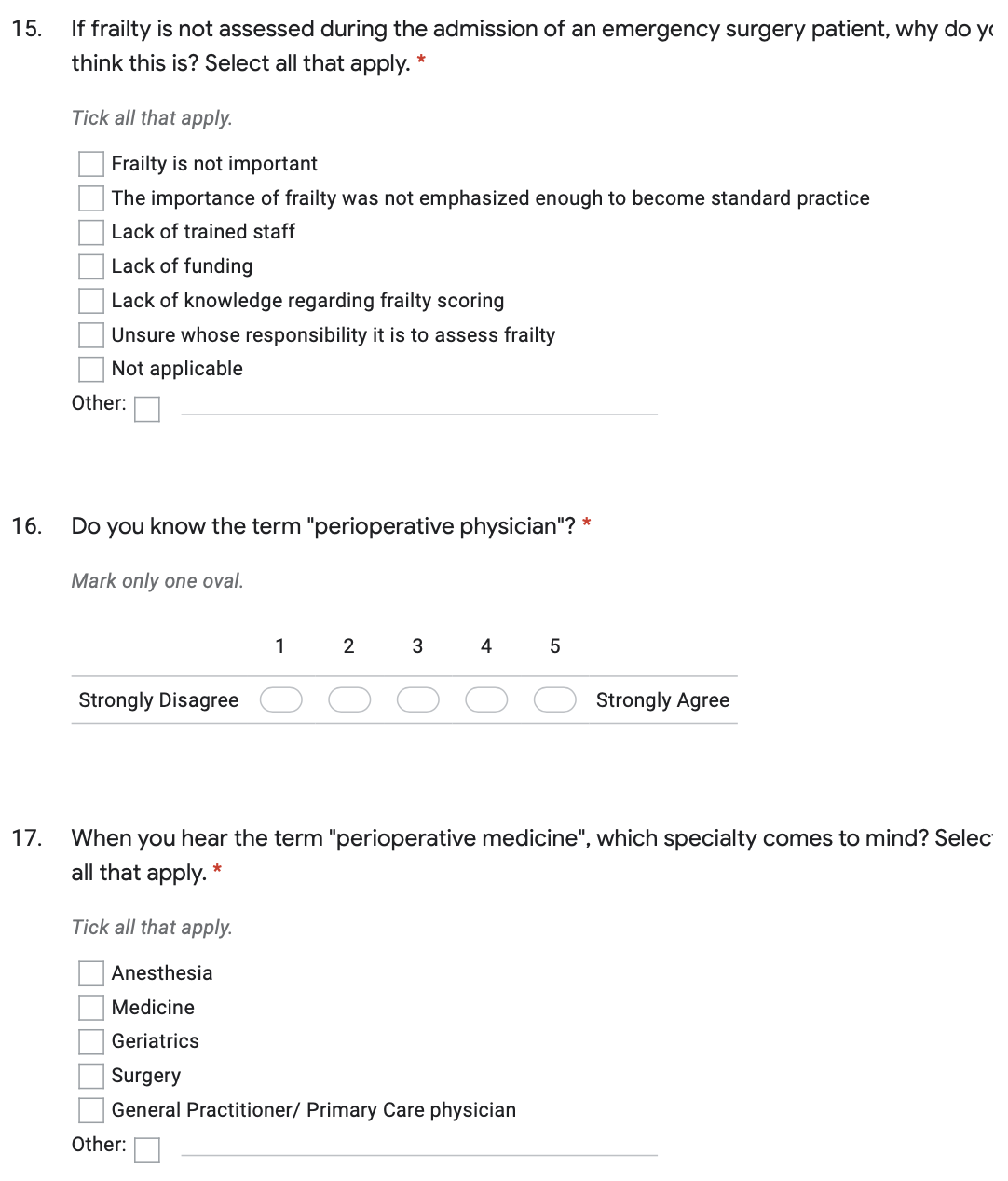

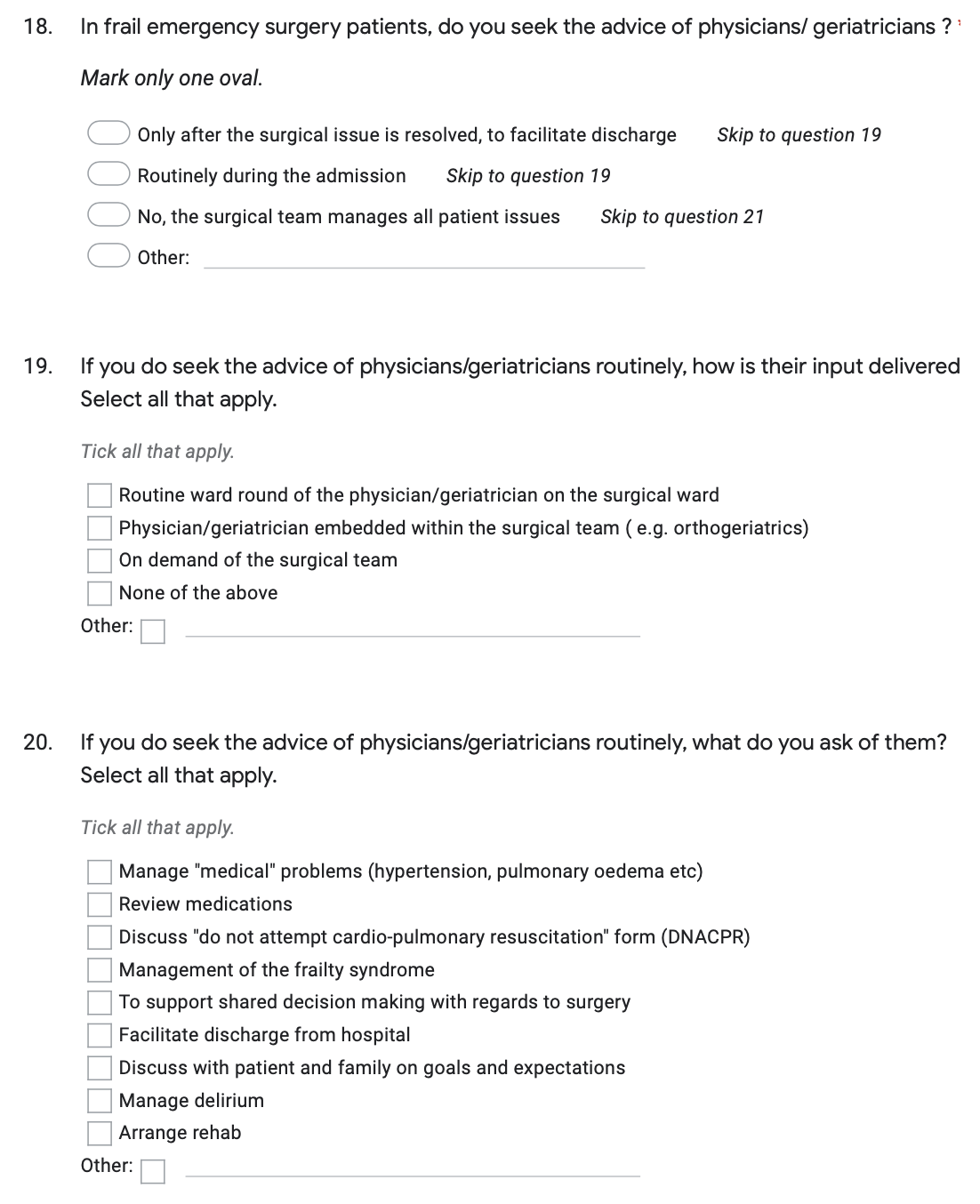

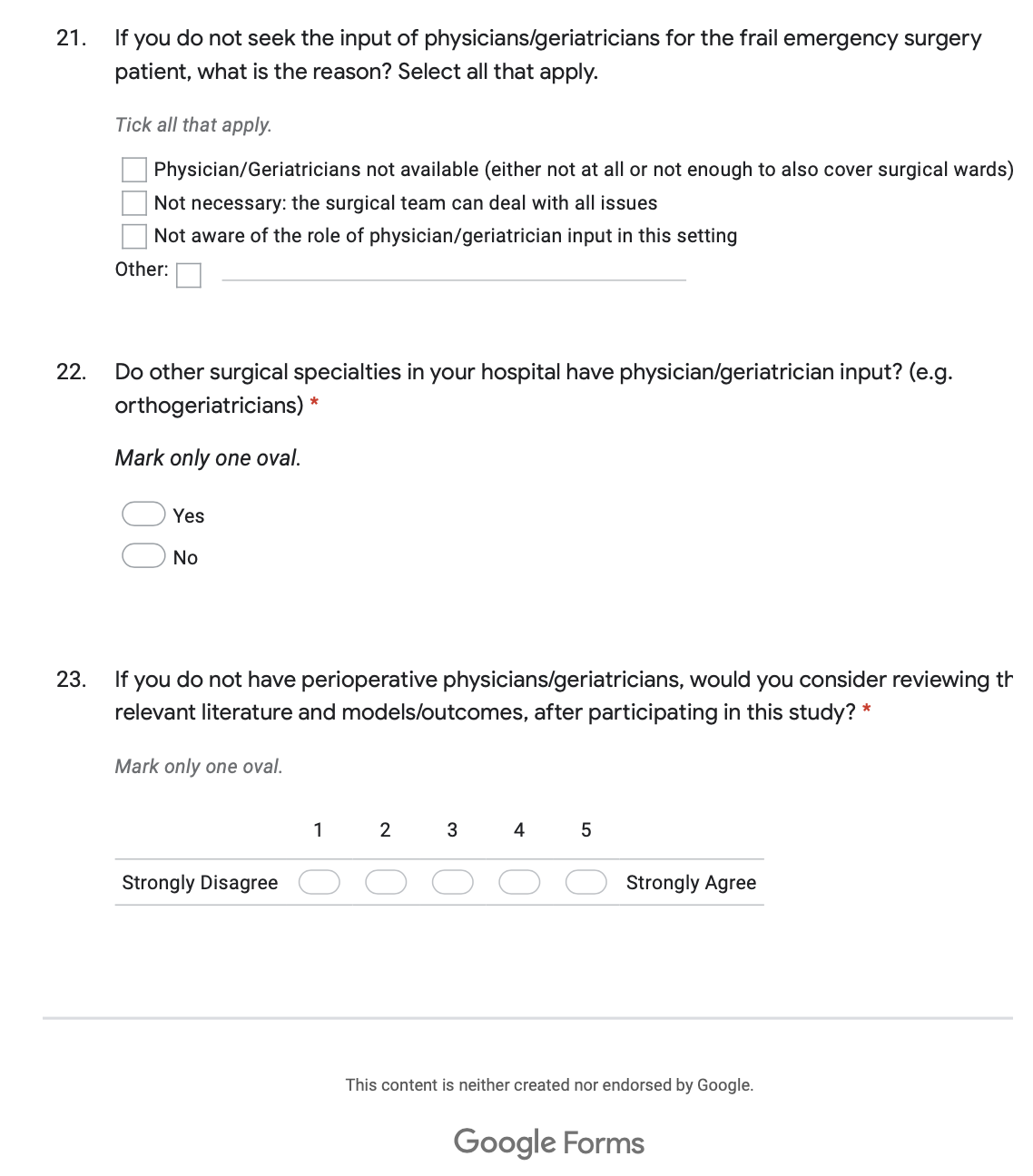
**

**Appendix (ii)**

Supplementary data

***Figure 1*** *shows whether the use of Risk Stratification tools varies by type of hospital*

***Figure 2*** *shows respondents’ awareness of the terms POPS and CGA by country and type of hospital.*
